# RETRACTION: HO‐1 Upregulation Attenuates Adipocyte Dysfunction, Obesity, and Isoprostane Levels in Mice Fed High Fructose Diets

**DOI:** 10.1155/jnme/9842407

**Published:** 2026-03-27

**Authors:** Journal of Nutrition and Metabolism

RETRACTION: Z. Khitan, M. Harsh, K. Sodhi, J. I. Shapiro, N. G. Abraham, “HO‐1 Upregulation Attenuates Adipocyte Dysfunction, Obesity, and Isoprostane Levels in Mice Fed High Fructose Diets,” *Journal of Nutrition and Metabolism*, 2014, 980547, https://doi.org/10.1155/2014/980547.

The above article, published online on 09 September 2014 in Wiley Online Library (https://wileyonlinelibrary.com), has been retracted by agreement between the journal editorial board member, Karen Sweazea; and John Wiley & Sons Ltd. The retraction has been agreed due to concerns that have been raised with the data, both directly and through PubPeer [1].

Specifically, concerns regarding the SEM and error bars in multiple figures of the article have been raised, and the authors were unable to provide the raw data. As such, the Editorial Board has concluded that the results and conclusions can no longer be considered reliable. The article is therefore retracted.

Zeid Khitan agrees with the retraction. The remaining authors were unresponsive.
